# The Crucial Findings Derived from the Special Issue “Inside Cancer Genomics: From Structure to Therapy”

**DOI:** 10.3390/cancers15133488

**Published:** 2023-07-04

**Authors:** Vincenza Barresi

**Affiliations:** Department of Biomedical and Biotechnological Sciences, University of Catania, 95123 Catania, Italy; vincenza.barresi@unict.it

Cancer initiation, growth, and progression are sustained by multiple types of genetic alterations, ranging in size from single point mutations, focal genomic errors to broad chromosomal copy number alterations, gains, and losses [1,2,3]. The identification of cancer-driving coding and non-coding elements located in structural variant regions is particularly difficult, since many genes and/or regulatory elements could be potentially involved [4,5,6,7]. The current extensive knowledge on DNA mutations in cancer genomes, derived from the application of omics technologies characterizing entire cancer genomes at structural and transcriptional levels, provides important insights into cancer’s molecular mechanisms, and inspires a vast range of targeted therapies [8,9,10]. In this sense, the correlation between a higher hCTR1 expression and higher platinum drug uptake in cancer cells sensitive to the drug has been reported [11,12] and the overexpression of copper transporter, CTR1, suggests a high sensitivity of CRC to platinum drugs [13].

The study of the influence of broad copy number aberrations detected by SNP-arrays on the transcriptome profile, analyzed by microarrays and RNA-seq technologies, revealed a high-density of upregulated genes in the chromosomes affected by gains, or, on the contrary, downregulated genes in the chromosomes affected by losses. Uri Ben-David and Angelika Amon discuss in detail the effect of aneuploidy to promote or suppress tumorigenesis and describe how metabolism, microenvironment, and immune system interactions can determine a fitness advantage for cancer development and progression under cell-type-specific conditions [14]. The analysis of gene-dosage transcriptional effects and the overexpression of cancer genes in selected altered recurrent chromosomes, such as Chr20, Chr 8, Chr13, and Chr7, have allowed the identification of crucial genes that cooperate at a functional level as subunits of protein complexes or in the same pathways sustaining cancer cell growth and progression. The authors found an increased expression of two genes encoding the eukaryotic translation initiation factors EIF3E and EIF3H, located in Chr8q, and a gene located in Chr20 and encoding the eukaryotic translation initiation factor EIF2S2, involved in the EIF2 signaling pathway of the initiation phase of mRNA translation [15,16]. Another significant pathway identified through the analysis of over-expressed transcripts and fitness-overexpressed transcripts (please see papers 15,16 for details) is the “Cleavage and Polyadenylation of Pre-mRNA”, which contains multiple amplified subunits belonging to this pathway. On the other hand, growing evidence indicates the implication of germline variations in cancer predisposition and prognostication.

The Special Issue, comprising 10 research articles and 3 reviews, aims to provide the latest findings on the above topics, with a particular focus on their potential biological relevance, the identification of predictive and prognostic biomarkers, and their therapeutic implications in solid and hematological malignancies. These updates highlight new opportunities for the management of cancer patients from the diagnosis to the prognosis and treatment, and require a deep knowledge of the structure of the genes and genome, the novel loss and gain of functions derived from the acquisition of alterations at the level of a single/few nucleotide(s), or extensively to several genes involved in the loss or the gain of chromosome p/q arms in the evolution and progression of cancer disease. The impact of recurrent cytogenetic aberrations of chromosome 1 and 16 on the transcriptome has been widely discussed in breast cancer by Privitera et al. [17]. In this paper, genomic and transcriptome analysis has been conducted in over 1000 breast samples belonging to The Cancer Genome Atlas (TCGA) in order to disentangle the contribution of the transcriptome among the different 1,16-chromogroups of breast cancer. The analysis, integrated with functional pathway analysis, has suggested the cooperation of overexpressed 1q genes and underexpressed 16q genes in the genesis of both ductal and lobular carcinomas, and has highlighted the putative role of genes encoding gamma-secretase subunits (APH1A, PSEN2, and NCSTN) and Wnt enhanceosome components (BCL9 and PYGO2) in 1q, and the glycoprotein E-cadherin (CDH1), the E3 ubiquitin-protein ligase WWP2, the deubiquitinating enzyme CYLD, and the transcription factor CBFB in 16q. The classification in 1,16-chromogroups has been a pivotal strategy with far-reaching implications for the selection of cancer cell models and novel experimental therapies. 

Mosquera Orgueira et al. [18] reported a significant enrichment of rare germline variations in two genes coding proteins involved in the regulation of oxidative stress pathways (*CHMP6* and *GSTA4*) in a cohort of 726 B-cell lymphoid neoplasm patients. The authors detected 1675 possible disrupting variants in genes associated with cancer, of which 44.75% were novel events and 7.88% were protein-truncating variants. Among these, the most frequently affected genes were *ATM*, *BIRC6*, *CLTCL1A*, and *TSC2*. Germline–germline or germline–somatic “double-hit” events were identified in cancer driver genes. Homozygous germline double-hit events were detected in 28 cases, and, surprisingly, six genes affected more than one patient, including the Hedgehog signaling gene *GLI1* and the homeobox tumor suppressor *ZFHX3*. Germline- somatic “double hit” events were observed in 17 patients, some of which affected key common lymphoma protein drivers such as ATM, KMT2D, MSH6, the histone acetyltransferase EP300, the histone gene HIST1H1E, the transcriptional regulator SIN3A, the NOTCH pathway member SPEN and the apoptotic proteins GNA13 and MCL1. As reported by the same authors, further research would be useful in order to overcome the heterogeneity of B-cell lymphoma disease. 

Park Ji et al. [19] reported, in a unique Korean pancreatic cancer patient cohort, that patients carrying germline BRCA1/2 mutations showed a higher response rate to platinum-based FOLFIRINOX therapy. The authors also showed that FOLFIRINOX chemotherapy administration prolonged (not significantly) the progression-free survival of BRCA-mutated patients. They finally concluded that a high proportion of germline BRCA1/2 mutations support the preferential use of FOLFIRINOX therapy for BRCA patients and the clinical utility to schedule early germline genetic testing.

Van de Velve et al. [20] focused their attention on the side effects of vincristine (VCR). This anti-cancer drug belongs to a group of compounds known as the vinca alkaloids, and it exerts its cytotoxic effect to prevent mitosis by interfering with microtubule polymerization. These types of drugs are frequently used for the treatment of several types of pediatric malignancies. Unfortunately, these treatments cause peripheral neuropathy in a subgroup of patients. Van de Velve et al. identified and replicated the genetic variants associated with VCR pharmacokinetics and neuropathy. Nine SNPs in seven genes were associated with VIPN and/or PK, of which four genes were related to the cytoskeleton (*CEP72*, a gene encoding a centrosomal protein required for adequate chromosome segregation, *SEPTIN9*, a gene that encodes a protein involved in cytokinesis and cell cycle control via the microtubules, *FIG4* and *FGD4*, genes that are both involved in the regulation of the actin cytoskeleton and cell shape); other polymorphisms have been found in genes involved in DNA expression and repair (SNU13 in Spliceosome and *ETAA1* in DNA repair). The association between genetic polymorphisms and VCR PK and VIPN in children with cancer provides insights that may prove useful in optimizing the VCR treatment of children with cancer.

Oparina et al. [21] have presented an interesting paper regarding the prognostic significance of BIRC5/survivin expression in three independent cohorts of breast cancer patients. Firstly, the authors analyzed the West Swedish Breast Cancer cohort (845 samples) and secondly, validated their findings in two independent cohorts, the Molecular Taxonomy of Breast Cancer International Consortium (METABRIC, 1939 samples, microarray data) and the population-based multicenter Sweden Cancerome Analysis Network-Breast Initiative (SCAN-B, 3073 samples, RNAseq data). The authors suggested that BIRC5/surviving mRNA and/or protein is a sensitive single marker of survival probability in breast cancer (BC), acting independently of ER and the nodal status of patients, and concluded that an analysis of its expression is useful when making treatment decisions in BC patients. Remarkably, BC tumors with undetectable protein survivin/BIRC5 had significantly better survival probability after 5 and 27 years. These data offer the opportunity for a new biomarker, BIRC5, and highlight the significance of the evaluation of BIRC5 levels in order to remodulate the intensity of cytotoxic treatment in those BC patients. For these reasons, this study should be of great interest for clinical oncologists and breast cancer researchers.

Priskin et al. [22] addressed an important question regarding routine laboratory activities and tested a self-developed NGS panel in a prospective pilot study of high-risk breast cancer patients in the environment of routine practice. The panel covers 38 regions of 12 genes (*AKT1*, *CDH1*, *EGFR*, *ESR1*, *FGFR1*, *156 FGFR4*, *HER2*, *HRAS*, *KRAS*, *MET*, *PIK3CA*, *TP53*), selected on the basis of their high frequency in breast cancer, for their predictive or prognostic value, to guide targeted treatment, or underlie the most frequent resistance cases. The tool “BC-monitor” could be useful to pre-determine cancer progression months earlier than conventional methods and support the treatment decision. After analyzing 44 patients, the authors speculated that the most important benefit of the use of the tool “BC-monitor” in the metastatic setting is its ability to assess, during real-time, the mutations that evolved during disease progression.

The development of transgenic and knockdown murine genetic models provides essential evidence regarding the importance of tumor suppressors. In relation to this, Vadakekolathu et al. [23] focused their attention on two p53-mutants, either R175H or R273H, in order to characterize their ability to acquire or lose novel oncogenic functions that directly influence protein–protein interactions in different ways. The authors performed multi-omics analyses on two P53 mutants, either R175H or R273H, by transfecting TP53 null osteosarcoma cells and SaOS-2, and identified several targets of immunotherapy. The phenotypic and functional changes, proliferation, gene expression, and quantitative SWATH proteomics combined with immune-peptidome analysis of the class-I eluted peptides identified several epitopes presented on the major histocompatibility complex (MHC). An in-silico analysis shortlisted which antigens were expressed in a range of cancerous tissues, but not the adjacent healthy ones. From the shortlist, 10 protein candidates were examined for their expression in 97 healthy tissues and 20 different tumor types belonging to the Human Protein Atlas. The DNA Topoisomerase II Alpha (TOP2A) and the microtubule-associated force-producing protein probably involved in organelle transport (KLC1) were the most frequently over-expressed of the upregulated proteins in SaOS mutants, and were found to be over-expressed in many cancers.

Arbel Rubinstein et al. [24] studied the role of klotho as a tumor suppressor in pancreatic ductal adenocarcinoma (PDAC). They employed a novel genetic model, combining pancreatic klotho knockdown with a mutation in Kras, and revealed that the lack of klotho contributed to PDAC development and decreased mouse survival. Klotho is an anti-aging transmembrane protein, which can be shed and can function as a hormone. The literature reports that klotho is a tumor suppressor in a wide array of malignancies, and indicates that the subdomain KL1 is responsible for this activity. This study demonstrates that the administration of viral particles carrying a spliced klotho isoform containing the KL1 domain (sKL) inhibited pancreatic tumors in a xenograft model, and that the treatment with soluble sKL prolonged the survival of a mouse model known to recapitulate human PDAC.

In the past few years, many studies have aimed to exponentially expand the characterization of cancer driver genes and mutations, oncogenic signaling pathways, and the amounts of transcripts that drive childhood and adult cancers. Along with advancements in high-throughput omics technologies, the construction of databases such as “The Cancer Genome Atlas” (TCGA https://www.cancer.gov/ccg/research/genome-sequencing/tcga) and “Genomic Data Commons Data Portal” (GDC https://portal.gdc.cancer.gov/), or “Therapeutically Applicable Research to Generate Effective Treatments (TARGET https://www.cancer.gov/ccg/research/genome-sequencing/target) and computational algorithms have been implemented to characterize genome and transcriptional diversity and to guide the development of more effective, tailored, and less toxic therapies [14,25,26,27]. Multiple genes in the human genome, when mutated, lead to the expansion and progression of tumors. Oncogenes are genes that, when mutated, produce molecules with a gain-of-function (GOF) that allows them to contribute to the dysregulation of the cell. On the contrary, tumor suppressor genes lose their ability to protect the cell from dysregulated growth and proliferation when they are mutated. In this regard, Funkhouser and colleagues [28] evaluated the potential role of serum levels of galectins -1, -3, -7, -8, and -9 as diagnostic and prognostic biomarkers in breast and non-small-cell lung cancer patients and their association with the mutation status of 50 oncogenes and tumor suppressor genes. They used a next generation sequencing (NGS) panel (Ion Ampliseq Cancer Hotspot Panel, v2, Life Technologies Corporation, Carlsbad, CA, USA), which covered approximately 2800 mutations reported in the Catalogue of Somatic Mutations in Cancer (COSMIC) database. Galectins (formerly known as S-type lectins), a family of lectin proteins, are the main players that trigger immune responses and resolve inflammation but they are also involved in other functions as they interact with cellular proteins, via binding to protein glycosylation sites, enhance oncogenic signals, and promote proliferation. Increased levels of galectins have been implicated in cancer progression, metastasis, and angiogenesis, and altered serum levels of galectins have been found in cancer cases. The authors performed the ELISA assay and found that high levels of galectin-1, -3, -8, and -9 are significantly associated with *KIT* mutations in breast cancer samples, and that high levels of galectin-1 and -7 are significantly associated with *PTEN* and *KIT* mutations in lung cancer samples. In the same Special Issue, another research group, Hu et al. [29], revealed that another tumor suppressor gene, *CSMD1*, is under-expressed in esophageal squamous cell carcinoma (ESCC). Its underexpression has been demonstrated in the recent literature in different tumors including lung, head and neck, breast, skin, colorectal, gastric, and ovarian cancers. The authors were the first to perform an integrative analysis to study the somatic DNA copy number alterations, expression at the level of mRNA, and targeted miRNA expression in the same ESCC patients and samples. Interestingly, they found *CSMD1* DNA alterations in two thirds of ESCC patients even if, due to tumor regional heterogeneity, the *CSMD1* exhibited considerable heterogeneity in tumor DNA alterations and expression levels when patients were grouped by the presence/absence of these alterations. The tumoral heterogeneity is an important issue that researchers have been trying to investigate in order to understand the biology of tumors, its effects on neoplastic transformation, and its relevance to cancer therapeutics [30,31,32].

The overexpression of proteins detected in cancer can mirror a condition of a protein synthesized in normal tissue and upregulated in cancer, or a protein activated only in a tumor and undetectable in normal tissue. To date, the functional role is not known for all proteins detected in a specific tissue. This is the case for SPINK2, an inhibitor of serine protease overexpressed in a subset of pediatric acute myeloid leukemias, and a hypothesis on its role in bone marrow has been only recently reported [33].

To date, more than one hundred different types of RNA modification have been described and numerous studies on RNA methylation have been performed since 2012. The methylation of different RNA species has emerged as a critical controller of expression. RNA methylation and its related downstream signaling pathways are involved in a lot of biological processes, including cell differentiation, stress response, and others. The role of methyladenosine modification has expanded from tumor genesis and metastasis to the regulation of the tumor microenvironment, immunotherapy, and drug resistance. Worthy of note is the contribution to the Special Issue of the recent findings that highlight the importance of methyladenosine modification in RNAs, from its regulatory activity to its therapeutic relevance in cancer. Qu et al. [34] describe methyladenosine modifications; the structures of m6A, m1A, and m6Am in eukaryotic cells; their generation; as well as the functions of writers, erasers, and readers with a focus on cancers. The review also covers their theragnostic opportunities.

In another review, Cetraro et al. [35] focused their attention on “The Inhibitors of Apoptosis Protein Family (IAPs)” as a potential pharmacological target in cancer, considering that in several cancers, the abnormal expression of IAPs can lead to dysregulated cell suicide. In addition, their natural antagonist, second mitochondrial-derived activator of caspases (Smac), appears to be downregulated and potentially correlated to the acquisition of resistance to traditional chemotherapy. Their review has been focused on the possible explanation of these effects, and on new findings that may contribute to the understanding of this phenomenon. Furthermore, the current state of combination therapy involving Smac mimetics with traditional agents, as well as with immunotherapy, has been presented.

Cinque et al. [36] summarize, in a review, the clinical spectrum and molecular features of an autosomal dominant inherited cancer syndrome, Von Hippel-Lindau (VHL) disease, in order to highlight new molecular opportunities based on genetic alterations, biological pathways, and potential biomarkers.

In conclusion, this Special Issue provides an overview of some recent advancements related to the structures, chemical modifications, and alterations present at the gene, transcript, protein, and genome levels, and their effects on cancer, from basic science to applications for clinical use.
